# Frailty Screening is Associated with Hospitalization and Decline in Quality of Life and Functional Status in Older Patients with Inflammatory Bowel Disease

**DOI:** 10.1093/ecco-jcc/jjad175

**Published:** 2023-10-23

**Authors:** Vera E R Asscher, Mar Rodriguez Gírondo, Jesse Fens, Sanne N Waars, Rogier J L Stuyt, A Martine C Baven-Pronk, Nidhi Srivastava, Rutger J Jacobs, Jeoffrey J L Haans, Lennart J Meijer, Jacqueline D Klijnsma-Slagboom, Marijn H Duin, Milou E R Peters, Felicia V Y L Lee-Kong, Nanda E Provoost, Femke Tijdeman, Kenan T van Dijk, Monse W M Wieland, Mirre G M Verstegen, Melissa E van der Meijs, Annemijn D I Maan, Floor J van Deudekom, Andrea E van der Meulen-de Jong, Simon P Mooijaart, P W Jeroen Maljaars

**Affiliations:** Department of Gastroenterology and Hepatology, Leiden University Medical Centre, Leiden, the Netherlands; Department of Biomedical Data Sciences, Leiden University Medical Center, Leiden, the Netherlands; Department of Gastroenterology and Hepatology, Leiden University Medical Centre, Leiden, the Netherlands; Department of Gastroenterology and Hepatology, Leiden University Medical Centre, Leiden, the Netherlands; Department of Gastroenterology and Hepatology, HagaZiekenhuis, The Hague, the Netherlands; Department of Gastroenterology and Hepatology, Groene Hart Ziekenhuis, Gouda, the Netherlands; Department of Gastroenterology and Hepatology, Haaglanden Medical Centre, The Hague, the Netherlands; Department of Gastroenterology and Hepatology, Alrijne Hospital, Leiden and Leiderdorp, the Netherlands; Department of Gastroenterology and Hepatology, Maastricht University Medical Centre, Maastricht, the Netherlands; Department of Gastroenterology and Hepatology, Leiden University Medical Centre, Leiden, the Netherlands; Department of Gastroenterology and Hepatology, Leiden University Medical Centre, Leiden, the Netherlands; Department of Gastroenterology and Hepatology, Leiden University Medical Centre, Leiden, the Netherlands; Department of Gastroenterology and Hepatology, Leiden University Medical Centre, Leiden, the Netherlands; Department of Gastroenterology and Hepatology, Leiden University Medical Centre, Leiden, the Netherlands; Department of Gastroenterology and Hepatology, Leiden University Medical Centre, Leiden, the Netherlands; Department of Gastroenterology and Hepatology, Leiden University Medical Centre, Leiden, the Netherlands; Department of Gastroenterology and Hepatology, Leiden University Medical Centre, Leiden, the Netherlands; Department of Gastroenterology and Hepatology, Leiden University Medical Centre, Leiden, the Netherlands; Department of Gastroenterology and Hepatology, Leiden University Medical Centre, Leiden, the Netherlands; Department of Gastroenterology and Hepatology, Leiden University Medical Centre, Leiden, the Netherlands; Department of Gastroenterology and Hepatology, Leiden University Medical Centre, Leiden, the Netherlands; Department of Gerontology and Geriatrics, Leiden University Medical Centre, Leiden, the Netherlands; Department of Gastroenterology and Hepatology, Leiden University Medical Centre, Leiden, the Netherlands; Department of Gerontology and Geriatrics, Leiden University Medical Centre, Leiden, the Netherlands; Department of Gastroenterology and Hepatology, Leiden University Medical Centre, Leiden, the Netherlands

**Keywords:** Crohn’s disease, ulcerative colitis, elderly, geriatric screening, functional decline

## Abstract

**Background and Aims:**

Our goals were to study frailty screening in association with hospitalization and decline in quality of life [QoL] and functional status in older patients with inflammatory bowel diseases [IBD].

**Methods:**

This was a prospective multicentre cohort study in IBD patients ≥65 years old using frailty screening [G8 Questionnaire]. Outcomes were all-cause, acute, and IBD-related hospitalization, any infection, any malignancy, QoL [EQ5D-3L], and functional decline (Instrumental Activities of Daily Living [IADL]) during 18 months of follow-up. Confounders were age, IBD type, biochemical disease activity [C-reactive protein ≥10 mg/L and/or faecal calprotectin ≥250 µg/g], and comorbidity [Charlson Comorbidity Index].

**Results:**

Of 405 patients, with a median age of 70 years, 196 [48%] were screened as being at risk for frailty. All-cause hospitalizations occurred 136 times in 96 patients [23.7%], and acute hospitalizations 103 times in 74 patients [18.3%]. Risk of frailty was not associated with all-cause (adjusted hazard ratio [aHR] 1.5, 95% confidence interval [CI] 0.9–2.4), but was associated with acute hospitalizations [aHR 2.2, 95% CI 1.3–3.8]. Infections occurred in 86 patients [21.2%] and these were not associated with frailty. A decline in QoL was experienced by 108 [30.6%] patients, and a decline in functional status by 46 patients [13.3%]. Frailty screening was associated with a decline in QoL (adjusted odds ratio [aOR] 2.1, 95% CI 1.3–3.6) and functional status [aOR 3.7, 95% CI 1.7–8.1].

**Conclusions:**

Frailty screening is associated with worse health outcomes in older patients with IBD. Further studies are needed to assess the feasibility and effectiveness of its implementation in routine care.

## Graphical Abstract

**Graphical Abstract:**
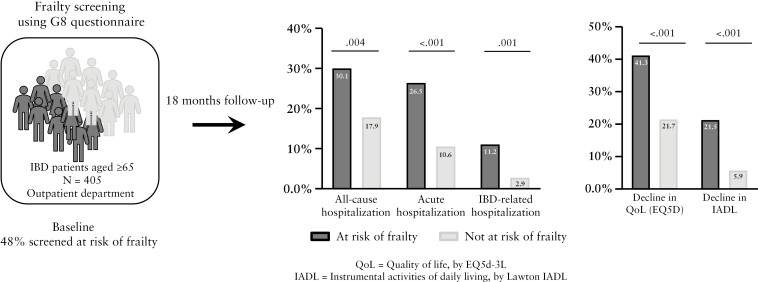

## 1. Introduction

Inflammatory bowel diseases [IBD], comprising Crohn’s disease [CD] and ulcerative colitis [UC], are chronic, relapsing–remitting inflammatory diseases affecting the gastrointestinal tract. The number of older patients with IBD is rising.^1^ Although it has been advised to assess an individual’s frailty when making treatment decisions in older patients with IBD, evidence on frailty and its association with outcomes in IBD is still scarce.^2,3^

A geriatric assessment includes an assessment of frailty and functioning in four domains [somatic, functional, mental, and social]. Comprehensive geriatric assessment, which includes geriatric assessment and an integrated care plan and follow-up, has been proven to be effective in improving outcomes of older patients with acute disease^4^ and older patients with cancer.^5^ Screening for risk of frailty, using validated instruments such as the Fried frailty phenotype^6^ or the Geriatric 8 questionnaire,^7^ however, could be more feasible in clinical practice as compared to a geriatric assessment.

In the field of IBD, little evidence is available on the association between frailty and negative health outcomes or functional status and [health-related] quality of life ([HR]QoL) over time.^3^ We recently published a study demonstrating an association between presence of deficits in geriatric assessment and higher disease burden and disease activity in older patients with IBD.^8^ It has also been recognized that frailty can occur in younger patients with IBD^9^ and that retrospectively assessed frailty is associated with negative health outcomes such as infections or hospital admissions in IBD patients.^10–12^ However, these latter longitudinal studies use frailty screening methods based on International Classification of Diseases [ICD] coding in administrative databases. Although using ICD coding could be appropriate for large-scale cohorts, it is difficult to translate to an individual patient level and is not suitable to aid complex clinical decision-making in daily practice.

The aim of the present study is to prospectively research the predictive value of frailty screening in older patients with IBD by assessing its association with hospitalization and decline in QoL and functional status.

## 2. Methods

### 2.1. Study design and patient population

This is a prospective multicentre cohort study performed in the outpatient departments and day treatment centres of six hospitals in the Netherlands, as previously described in detail.^8^ Patients were asked to participate during their regular hospital visit. Baseline visits took place between November 2016 and February 2020. Inclusion criteria were an age of 65 years or older and a confirmed clinical, endoscopic, and/or histological diagnosis of CD, UC, or IBD-Unclassified [IBD-U]. Patients unable or unwilling to participate, sign informed consent, or unable to speak Dutch or English were excluded. The Strengthening the Reporting of Observational studies in Epidemiology [STROBE] guidelines were followed.^13^

### 2.2. Data collection at baseline

Baseline data were collected face-to-face and were verified using the electronical medical record, as previously described.^8^ Demographic and IBD characteristics included age, sex, weight, height, disease type, disease duration, disease behaviour and location according to the Montreal classification^14^ [maximum extent at inclusion], current and previous IBD medications, and prior IBD-related surgery. Clinical disease activity was measured through the Harvey–Bradshaw Index [HBI] for CD patients^15^ and partial Mayo score [pMS]^16^ for UC or IBD-U patients. Active disease was defined by HBI > 4 or pMS ≥ 2. C-reactive protein [CRP] and faecal calprotectin [FCP] were extracted from the electronical medical record if tests were performed within 3 months of baseline. Biochemical disease activity was defined by either a CRP ≥ 10 mg/L or FCP ≥ 250 µg/g. To further specify biochemical disease activity, elevated FCP levels were reported separately as well. Endoscopic data were used if endoscopy was performed within 6 months of baseline.

The G8 questionnaire was used as a geriatric screening method.^7^ The G8 questionnaire consists of eight questions with a total score ranging from zero to 17; a score of ≤14 points indicates a risk of frailty. The G8 screening tool was developed in oncology patients^7^ and has also been validated in older adults without cancer.^17^

### 2.3. Data collection at follow-up

Patients were contacted for follow-up assessment either at their regular hospital appointment or by phone. During this contact, patients were asked about hospital admissions, infections, and malignancies during study period and these data were checked using the electronical medical record. Also, questionnaires regarding [HR]QoL and functional status were taken [see below]. Follow-up assessment was aimed to take place 18 months after baseline. The primary outcome was only noted if occurring within 18 months from baseline. For all patients who were not able to participate in follow-up contact, data regarding primary and secondary outcomes were extracted from the electronical medical record.

The primary outcome of this study was the occurrence of all-cause hospital admissions during 18 months of follow-up. Hospitalizations were further specified as acute or IBD-related hospitalizations. Acute hospitalizations were defined as all non-elective hospital admissions. The secondary outcome was the presence of infection during follow-up, which were noted as any infection or serious infection. All infections were noted when occurring between baseline and follow-up contact, or if the patient was lost to follow-up between baseline and 18 months after baseline. Serious infections were defined as an infection needing hospital admission. The occurrence of malignancies and mortality were also noted.

The tertiary outcome was a decline in [HR]QoL or functional status. HRQoL was assessed using the short Inflammatory Bowel Disease Questionnaire [sIBDQ],^18^ a questionnaire containing ten questions resulting in a score ranging from ten to 70 [a high score equals a high HRQoL]. A decline in QoL was measured with EQ-5D-3L, a standardized questionnaire on QoL developed by the Euroqol group^19^ using five health aspects and was scored using the Dutch value set to obtain index values standardized from 0 to 1; 0 represents death and 1 represents full health.^20^ A negative difference in HRQoL or QoL at follow-up as compared to baseline was considered a decline. A decline in functional status was measured using the Katz Index of Independence in Activities of Daily Living [ADL]^21^ and the Lawton Instrumental Activities of Daily Living [IADL].^22^ A decrease in ADL or IADL score of ≥1 was considered a decline.

### 2.4. Statistical analyses

Continuous variables are presented as mean with standard deviation [SD] or as median with interquartile range [IQR] and are compared using an independent t test or Mann–Whitney U test, depending on the normality of the distribution of the data. Categorical variables are presented as numbers and percentages and compared using a chi-square test or Fisher’s exact test. Time from baseline to first hospitalization was considered as an outcome and therefore a Kaplan–Meier method for description and Cox proportional hazards model for association between frailty and primary outcome was used. The proportional hazard assumption was checked by testing each variable’s interaction with time and visual inspection of the Schoenfeld residuals. Because for secondary outcomes no date of occurrence of infection was known, and for tertiary outcomes all patients were measured at the same time point [18 months after baseline], binary logistic regression analyses were used for these outcomes. Analyses were performed as complete case analyses. Potential confounders were agreed upon beforehand and included age at baseline [continuous variable], biochemical disease activity [elevated CRP and/or FCP, binary variable], comorbidity [Charlson Comorbidity Index (CCI), continuous variable] and IBD type [CD vs UC/IBD-U] for the association between frailty screening and primary and tertiary outcomes. Regarding the secondary outcome, baseline IBD medication [oral corticosteroid use, immunomodulator use, biological therapy] was added as a potential confounder. No sample size calculation was performed and we aimed to include as many patients as possible to create a representative cohort. A *p*-value of <0.050 was considered statistically significant. Data analyses were performed using IBM SPSS Statistics for Windows, version 25.

We estimated the predictive performance of the multivariate model to predict all-cause hospitalization. Discrimination was quantified by the C-index ranging from 0.5 [no discrimination] to 1.0 [perfect discrimination], internally validated by bootstrapping. Bootstrap analysis was performed using R, version 4.02.

### 2.5. Ethical considerations

The study protocol was declared not subject to the medical research involving human subjects act by the Committee on Research Involving Human Subjects at the LUMC and was approved in all participating centres. All patients provided written informed consent.

## 3. Results

At baseline, 547 patients were eligible for inclusion, 405 of whom were included in our study [for study flowchart see Supplementary Figure 1]. Baseline characteristics are listed in Table 1. The overall median age was 70 years [IQR 67–74], 188 patients were female [46.4%], and 191 patients [47.0%] were diagnosed with CD. Eighty-five patients [21.0%, 14 missing] had clinical IBD activity, 93 patients [23.0%, 57 missing] had biochemical disease activity [elevated CRP or FCP], and 68 patients [16.8%, 176 missing] had an elevated FCP. Frailty screening was performed using the G8 questionnaire; 196 patients [48.3%] were screened as being at risk of frailty. In Table 1 baseline characteristics are displayed by risk of frailty. Patients at risk of frailty were older [median 71.0 vs 70.0 years, *p* = 0.001], were more often female [55.1% vs 37.7%, *p* < 0.001], and had a higher percentage of clinical [29.6% vs 14.3%, *p* < 0.001] and biochemical [33.5% vs 19.9%, *p* = 0.004] disease activity.

**Table 1.** Baseline characteristics by risk of frailty.

	Total cohort [*n* = 405]	No risk of frailty [*n* = 207]	Risk of frailty [*n* = 196]	*p*-value
Median age at baseline, years [IQR]	70.0 [67.0–74.0]	70.0 [67.0–72.0]	71.0 [68.0–75.0]	0.001
Median disease duration, years [IQR]	22.0 [7.0–39.5]	19.0 [7.0–37.0]	25.0 [8.0–41.0]	0.055
Sex [female]	188 [46.4]	78 [37.7]	108 [55.1]	<0.001
Educational level [high]	121 [29.9]	64 [31.7]	57 [31.0]	0.881
Current smoker	39 [9.6]	23 [11.1]	16 [8.2]	0.317
IBD type				0.213
CD	191 [47.2]	89 [43.0]	101 [51.5]	
UC	202 [49.9]	112 [54.1]	89 [45.4]	
IBD-U	12 [3.0]	6 [2.9]	6 [3.1]	
Current ostomy				0.198
No ostomy	374 [92.3]	195 [94.2]	177 [90.3]	
Ileostomy	26 [6.4]	9 [4.3]	17 [8.7]	
Colostomy	5 [1.2]	3 [1.4]	2 [1.0]	
Older-onset IBD	136 [33.6]	70 [33.8]	65 [33.2]	0.890
Age at diagnosis, years				0.730
≤16	9 [2.2]	4 [1.9]	5 [2.6]	
17–40	147 [36.3]	72 [34.8]	74 [37.8]	
>40	249 [61.5]	131 [63.3]	117 [59.7]	
Disease location [CD]				0.193
Ileum	51 [26.7]	24 [27.0]	27 [26.7]	
Colon	35 [18.3]	21 [23.6]	14 [13.9]	
Ileocolonic	105 [55.0]	44 [49.4]	60 [59.4]	
Upper GI involvement [CD]	11 [5.8]	6 [6.7]	5 [5.0]	0.598
Disease behaviour [CD]				0.101
Inflammatory	79 [41.4]	44 [49.4]	35 [34.7]	
Stricturing	59 [30.9]	25 [28.1]	33 [32.7]	
Penetrating	53 [27.7]	20 [22.5]	33 [32.7]	
Peri-anal disease [CD]	46 [24.1]	22 [24.7]	24 [23.8]	0.878
Disease location [UC/IBD-U]				0.066
Proctitis	31 [14.5]	23 [19.5]	8 [8.4]	
Left-sided colitis	76 [35.5]	38 [32.2]	38 [40.0]	
Pancolitis	107 [50.0]	57 [48.3]	49 [51.6]	
Median CRP, mg/L [IQR]	3.0 [1.9–5.0]	3.0 [1.2–4.0]	3.0 [2.0–6.0]	0.001
Median FCP, µg/g [IQR]	119.0 [36.5–328.3]	82.0 [25.8–233.0]	141.5 [46.0–414.0]	0.007
Elevated FCP [>250 µg/g]	68 [16.8]	26 [23.6]	41 [35.0]	0.060
Biochemical disease activity [CRP ≥ 10 mg/L and/or CRP ≥ 250 µg/g]	93 [23.0]	35 [19.9]	57 [33.5]	0.004
Endoscopic disease activity	65 [16.0]	32 [45.7]	33 [46.5]	0.927
Clinical disease activity [HBI > 4 or pMS ≥ 2]	85 [21.0]	29 [14.3]	55 [29.6]	<0.001
Median HBI [IQR]	2.0 [1.0–4.0]	2.0 [1.0–3.0]	3.0 [1.5–5.0]	0.001
Median pMS [IQR]	0.0 [0.0–1.0]	0.0 [0.0–1.0]	1.0 [0.0–2.0]	0.009
Current IBD therapy				
No current IBD therapy	86 [21.2]	38 [18.4]	48 [24.4]	0.133
Mesalamine	170 [42.0]	92 [44.4]	78 [39.8]	0.345
Prednisone or budesonide	39 [9.6]	14 [6.8]	25 [12.8]	0.042
Immunomodulator	81 [20.0]	40 [19.3]	39 [19.9]	0.885
Biological	107 [26.4]	52 [25.1]	54 [27.6]	0.580
Prior IBD surgery	156 [38.5]	70 [33.8]	86 [43.9]	0.038

Valid percentages are reported; missing data: educational level, 17; CRP, 81; FCP, 176; biochemical disease activity, 57; endoscopic disease activity, 264; clinical disease activity, 14.

IQR, interquartile range; CD, Crohn’s disease; UC, ulcerative colitis; IBD-U, IBD-Unclassified; CRP, C-reactive protein; FCP, faecal calprotectin; HBI, Harvey–Bradshaw Index; pMS, partial Mayo Score; IBD, inflammatory bowel disease.

High educational level: higher vocational or university.

Only oral IBD therapy was noted.

For type of biologicals used see Supplementary Table 5.

We were able to contact 356 patients [87.9%] for follow-up questionnaires [see study flow-chart in Supplementary Figure 1]. Eleven patients died during follow-up, nine of whom were screened as frail at baseline [Supplementary Table 4]. Mean duration from baseline to follow-up contact was 560 days [IQR 546–614.5].

### 3.1. Outcomes

A total of 136 all-cause hospitalizations occurred during follow-up in 96 patients [23.7%]. Of all hospitalizations, 103 [75.7%] were acute, occurring in 74 patients [18.3%]. Forty-one hospitalizations [30.1%] in 28 patients [6.9%] were IBD-related [see Supplementary Table 1 for details on reasons for hospitalization].

**Figure 1.**
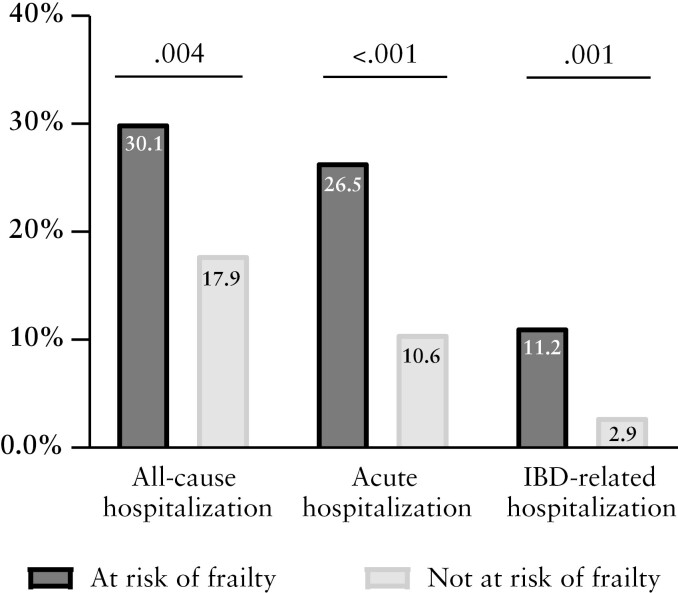
Hospitalizations during 18 months of follow-up in older patients with IBD by frailty screening. Percentage of patients hospitalized during 18 months of follow-up. Frailty screening was performed using the Geriatric 8 questionnaire. IBD, inflammatory bowel diseases.

**Figure 2.**
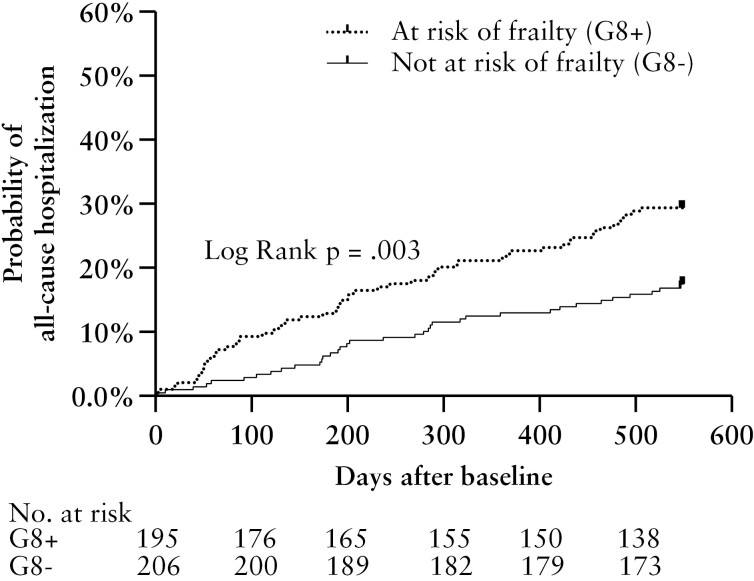
Probability of all-cause hospitalization according to frailty screening in older patients with inflammatory bowel disease. A Kaplan–Meier graph is displayed; frailty screening was performed using the Geriatric 8 questionnaire.

Patients at risk of frailty were more often hospitalized during follow-up for all-cause, acute, and IBD-related causes [Figures 1 and 2]. Risk of frailty was associated with all-cause acute hospitalizations and with IBD-related hospitalizations [Figure 1; Table 2]. Chronological age was not associated with primary outcome. No evidence against the proportional hazards assumption was found. The internally validated C-index of the prediction model for all-cause hospitalization was 0.651, indicating acceptable discriminatory ability.

**Table 2.** Risk of frailty and its association with all-cause hospitalization during follow-up, univariable and multivariable analyses.

	HR	95% CI	*p*–value	aHR	95% CI	*p*-value
**All-cause hospitalization**						
Risk of frailty	1.860	1.227–2.820	0.003	1.524	0.954–2.435	0.078
Age at baseline	1.006	0.966–1.047	0.784	0.967	0.925–1.011	0.139
Biochemical disease activity	2.482	1.616–3.812	<0.001	2.101	1.339–3.297	0.001
Comorbidity	1.340	1.174–1.529	<0.001	1.272	1.100–1.470	0.001
Crohn’s disease	1.225	0.818–1.836	0.325	1.049	0.683–1.614	0.826
**Acute hospitalization**						
Risk of frailty	2.859	1.722–4.746	<0.001	2.195	1.255–3.837	0.006
Age at baseline	1.020	0.976–1.066	0.386	0.982	0.937–1.030	0.464
Biochemical disease activity	2.765	1.710–4.471	<0.001	2.159	1.304–3.575	0.003
Comorbidity	1.451	1.259–1.673	<0.001	1.303	1.113–1.525	0.001
Crohn’s disease	1.411	0.890–2.238	0.143	1.158	0.712–1.883	0.555
**IBD-related hospitalization**						
Risk of frailty	4.069	1.650–10.036	0.002			
Age at baseline	1.040	0.972–1.113	0.253			
Biochemical disease activity	3.379	1.608–7.102	0.001			
Comorbidity	1.263	1.010–1.578	0.040			
Crohn’s disease	3.463	1.472–8.146	0.004			

Cox regression analyses. Analyses were performed as complete case analyses; 344 patients were included in multivariable analyses of all-cause hospitalizations [*n* = 169 at risk of frailty, *n* = 85 all-cause hospitalization], 345 patients in acute hospitalization multivariable analyses [*n* = 170 at risk of frailty, *n* = 67 acute hospitalization]. No multivariable analyses were performed for IBD-related hospitalization due to the small number of outcomes. Frailty screening by Geriatric 8 Questionnaire, ≤14 points = risk of frailty. Biochemical disease activity: C-reactive protein ≥10 mg/L and/or faecal calprotectin ≥250 µg/g. Comorbidity measured by Charlson Comorbidity Index, continuous.

### 3.2. Secondary outcomes

During follow-up 86 patients [21.2%] had any infection, 13 [15.1%] of whom needed hospitalization [see Supplementary Table 2 for details on infections]. Patients screened at risk of frailty did not have a higher infection rate [24.0% vs 18.4%, *p* = 0.167]. However, the rate of infections that needed hospitalization was higher in patients at risk of frailty (11 patients [5.6%] vs two patients [1.0%], *p* = 0.008).

Frailty screening [Table 3] was not associated with any infection during follow-up. Patients using biological therapy at baseline [*N* = 107 patients, 26.4%] had a higher risk of any infection during follow-up [Table 3]. Fifteen patients were diagnosed with a malignancy during follow-up, and eight of these had an abnormal frailty screening at baseline [Supplementary Table 3].

**Table 3.** Risk of frailty and its association with any infection during follow-up, univariable and multivariable analyses.

	OR	95% CI	*p*-value	aOR	95% CI	*p*-value
Risk of frailty	1.403	0.867–2.269	0.168	1.550	0.898–2.675	0.115
Age at baseline	0.963	0.915–1.014	0.154	0.964	0.911–1.020	0.205
Biochemical disease activity	1.013	0.574–1.789	0.964	0.859	0.460–1.605	0.634
Comorbidity	1.016	0.853–1.212	0.855	0.942	0.768–1.156	0.568
Crohn’s disease	1.226	0.761–1.974	0.403	0.857	0.486–1.511	0.593
Oral corticosteroid use	1.524	0.726–3.202	0.266	1.321	0.600–2.908	0.489
Immunomodulator use	0.810	0.436–1.504	0.505	0.691	0.349–1.370	0.290
Biological therapy	2.354	1.422–3.897	0.001	2.271	1.280–4.028	0.005

Logistic regression analyses. Analyses were performed as complete case analyses; 346 patients were included in multivariable analyses [*n* = 170 risk of frailty, *n* = 76 any infection]. Frailty screening by Geriatric 8 Questionnaire, ≤14 points = risk of frailty. Biochemical disease activity: C-reactive protein ≥10 mg/L and/or faecal calprotectin ≥250 µg/g. Comorbidity measured by Charlson Comorbidity Index, continuous.

### 3.3. Tertiary outcomes

At follow-up contact, we assessed the decline in QoL and functional status [Table 4].

**Table 4.** Risk of frailty and its association with patient-reported outcome measures during follow-up, univariable and multivariable analyses.

	OR	95% CI	*p*-value	aOR	95% CI	*p*-value
**Decline in QoL**						
Risk of frailty	2.546	1.597–4.058	0.000	2.129	1.258–3.605	0.005
Age at baseline	1.065	1.017–1.116	0.008	1.044	0.992–1.100	0.099
Biochemical disease activity	1.359	0.787–2.347	0.271	1.103	0.613–1.984	0.743
Comorbidity	1.155	0.977–1.365	0.091	1.033	0.854–1.249	0.736
Crohn’s disease	1.080	0.686–1.701	0.739	1.071	0.644–1.781	0.792
**Decline in HRQoL**						
Risk of frailty	1.293	0.838–1.996	0.245	1.582	0.958–2.611	0.073
Age at baseline	0.964	0.919–1.010	0.122	0.948	0.899–.999	0.047
Biochemical disease activity	0.655	0.379–1.133	0.130	0.620	0.345–1.115	0.111
Comorbidity	0.895	0.755–1.062	0.205	0.908	0.750–1.101	0.328
Crohn’s disease	0.820	0.531–1.265	0.369	0.727	0.449–1.178	0.195
**Decline in ADL**						
Risk of frailty	1.963	1.023–3.767	0.042	1.756	0.856–3.602	0.124
Age at baseline	1.007	0.943–1.075	0.836	0.990	0.921–1.065	0.794
Biochemical disease activity	0.847	0.383–1.875	0.682	0.823	0.356–1.903	0.648
Comorbidity	1.110	0.882–1.398	0.374	1.026	0.791–1.330	0.848
Crohn’s disease	1.934	1.008–3.710	0.047	1.739	0.863–3.504	0.122
**Decline in IADL**						
Risk of frailty	4.362	2.128–8.941	0.000	3.662	1.661–8.076	0.001
Age at baseline	1.077	1.015–1.142	0.014	1.036	0.970–1.107	0.295
Biochemical disease activity	1.898	0.941–3.830	0.073	1.411	0.653–3.046	0.381
Comorbidity	1.094	0.873–1.371	0.437	0.923	0.716–1.191	0.539
Crohn’s disease	0.986	0.529–1.839	0.965	0.911	0.446–1.860	0.799

Frailty screening by Geriatric 8 Questionnaire, ≤14 points = risk of frailty. Biochemical disease activity: C-reactive protein ≥10 mg/L and/or faecal calprotectin ≥250 µg/g. Comorbidity measured by Charlson Comorbidity Index, continuous. QoL = quality of life; measured with EQ5D-3L. HRQoL = health-related quality of life; measured with Short Inflammatory Bowel Disease Questionnaire. Functional decline measured with Katz Index of Independence in Activities of Daily Living [ADL] and the Lawton Instrumental Activities of Daily Living [IADL]. A decline in ADL or IADL score of ≥1 was considered a decline.

Logistic regression analyses were performed as complete case analyses. QoL: 298 patients included in analyses [*n* = 140 risk of frailty, *n* = 94 decline in QoL], HRQoL: 296 patients included in analyses [*n* = 140 risk of frailty, *n* = 119 decline in HRQoL], ADL: 297 patients included in analyses [*n* = 139 risk of frailty, *n* = 39 decline in ADL], IADL: 292 patients included in analyses [*n* = 137 risk of frailty, *n* = 39 decline in IADL].

QoL was measured based on EQ5D-3L in 353 patients at both baseline and follow-up; 108 of 353 patients [30.6%] experienced a decline in QoL. Risk of frailty was independently associated with a decline in QoL (adjusted odds ratio [OR] 2.129, 95% confidence interval [CI] 1.258–3.605, *p* = 0.005). HRQoL was measured by SIBDQ in 348 patients at both baseline and follow-up, and a decline in SIBDQ score was experienced in 135 patients [39.0%]. Risk of frailty was not associated with a decline in HRQoL.

A decline in functional status was measured by ADL and IADL. ADL was available for 351 patients at both baseline and follow-up; 43 patients [12.3%] showed a decline in ADL during follow-up, and the risk of frailty was not associated with a decline in ADL after correcting for confounders. IADL was available for 347 patients at both baseline and follow-up; 46 patients [13.3%] experienced a decline in IADL. Risk of frailty was independently associated with a decline in IADL [adjusted OR 3.662, 95% CI 1.661–8.076, *p* = 0.001].

## 4. Discussion

In the present study we found that frailty screening was independently associated with hospitalizations in older patients with IBD. Second, frailty screening was associated with a higher risk of decline in QoL and functional status over time.

The number of older patients with IBD is increasing due to both a rising prevalence and a rising incidence.^23,24^ In other research fields [such as rheumatology and hepatology], frailty, a state of increased vulnerability,^6,25^ can successfully function as a risk stratification tool in the treatment of older patients.^26–28^ In the IBD research field, the population of older patients is gaining increasing attention and several papers have called for action or described possible mechanisms between IBD and frailty.^3,29–31^ Recently, a number of studies have been published on the association between frailty screening and outcomes in patients with IBD. These studies provided evidence on the association between frailty screening and mortality,^10,32^ readmission,^10^ and infections in patients treated with immunosuppression.^11^ Singh et al. studied the association between frailty screening and serious infections in biologic-treated patients, but did not find an independent association.^33^ In these papers frailty was retrospectively screened for, using ICD codes or applying hospital frailty risk scoring systems in administrative databases.

In the present study we investigated the association between prospectively assessed frailty screening and negative health outcomes in older patients with IBD. An independent association was found between the risk of frailty and both all-cause and IBD-related hospitalization over time.

Risk of frailty was not associated with infections during follow-up, although patients at risk of frailty had more serious infections. In the above-mentioned study by Singh et al.^33^ in anti-TNF- and vedolizumab-treated patients with IBD of all ages the authors also found a higher rate of serious infections in frail patients, but after adjusting for confounders this risk was no longer significant.

Next, we researched the decline in [HR]QoL and functional status during follow-up. Risk of frailty was independently associated with a decline in both QoL and functional status. In other studies of older patients at the emergency department^34^ or in oncology,^35^ this association has already been established. In older patients, outcomes regarding functional status and QoL could be more important than established IBD-related outcomes such as mucosal healing.^36^

The important strengths of this study are its prospective nature and the inclusion of both referral and general hospitals. Furthermore, we used a validated frailty screening tool. Last, we included outcomes which are important in an older patient population, namely functional status and QoL.^3,37^ However, there are also some limitations. First, our results may be subject to ascertainment bias as frail patients, patients with [biochemical] disease activity, or patients treated with biologicals will have more contacts with their physician or scheduled hospital visits, and therefore more outcomes are noted in the electronical medical record. To limit this chance of bias, we not only checked the electronical medical record for outcomes but also planned follow-up contacts via phone or during regular hospital visits. Second, biochemical disease activity and endoscopic disease activity were not measured for study purposes. Therefore, these baseline data are not complete. However, by choosing to do so, we created a low barrier for patients to participate and were able to create a large and representative cohort. Furthermore, it is important to note that a frailty screening tool was used, which is designed to screen patients at risk of frailty who would benefit from further assessment by a comprehensive geriatric assessment.

Future studies should focus on developing a prediction model which could identify patients at risk for hospitalization or decline in [HR]QoL and functional status. A prediction model with the current data predicting all-cause hospitalization including frailty screening, biochemical disease activity, and comorbidity as predictors yielded a C-index of 0.653. The size of the current cohort and lack of a validation cohort made the development of a fully clinically applicable model less reasonable in our study.

Other research needs to focus on assessing frailty at multiple time points, assessing frailty and its predictive values in adult patients, and investigating the relationship between frailty and biochemical disease activity. A study by Lai et al.^38^ in patients with liver cirrhosis found that a worsening of frailty was significantly associated with death and delisting from the transplantation list, whereas patients with improvements in frailty had a lower risk of death or delisting. Besides, as frailty consists of modifiable elements such as nutritional status, depression and physical status, studies could focus on ameliorating frailty status, for example prior to the start of medical treatment. This concept is already being researched in surgery, for example prior to oesophagogastric cancer resection^39^ or colorectal surgery.^40^ Another option could be to select different therapy strategies in frail patients as compared to fit older patients with IBD to minimize negative health outcomes.

In conclusion, the findings of this paper emphasize the importance of assessing frailty in older patients with IBD. Frail patients are at a greater risk for both hospitalization and a decline in QoL and functional status. Future studies should focus on implementation of frailty assessment in routine care and the effectiveness of interventions to improve outcomes in older frail patients.

## Supplementary Material

jjad175_suppl_Supplementary_Figure_S1

jjad175_suppl_Supplementary_Table_S1-S5

## Data Availability

Data are available upon reasonable request.
