# Supplementary figures and images for: Frailty Screening is Associated with Hospitalization and Decline in Quality of Life and Functional Status in Older Patients with Inflammatory Bowel Disease
Source: J Crohns Colitis. 2023 Oct 23;18(4):516–24. doi: 10.1093/ecco-jcc/jjad175 (PMC11037105; doi:10.1093/ecco-jcc/jjad175)

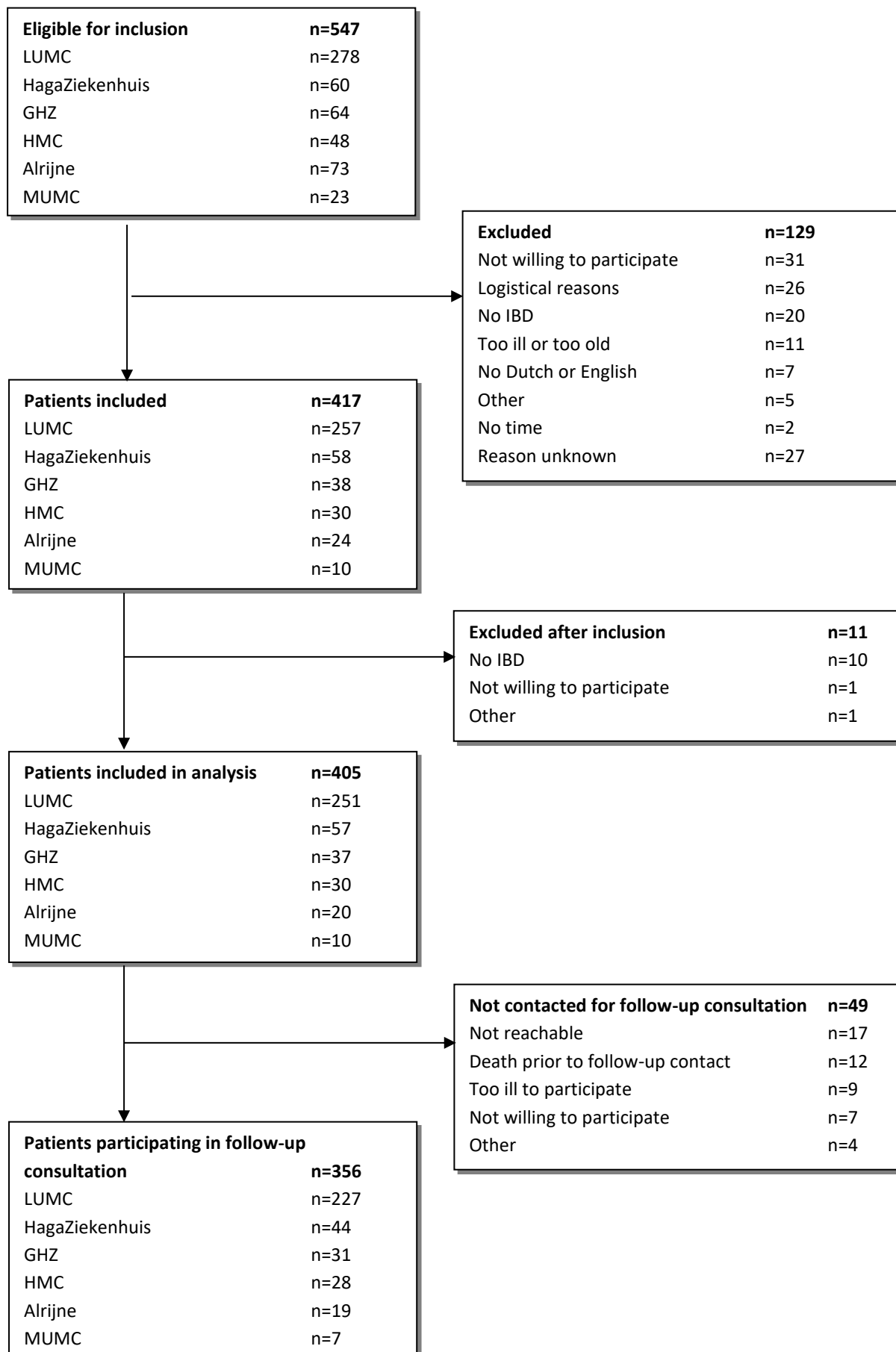

Supplement: jjad175_suppl_Supplementary_Figure_S1 [file jjad175_suppl_supplementary_figure_s1.pdf]
