# Supplementary material for: Frailty Screening is Associated with Hospitalization and Decline in Quality of Life and Functional Status in Older Patients with Inflammatory Bowel Disease
Source: J Crohns Colitis. 2023 Oct 23;18(4):516–24. doi: 10.1093/ecco-jcc/jjad175 (PMC11037105; doi:10.1093/ecco-jcc/jjad175)
Supplement: jjad175_suppl_Supplementary_Table_S1-S5 [file jjad175_suppl_supplementary_table_s1-s5.docx]

Supplementary figure 1. Study flowchart.
Flowchart patient inclusion.
Logistical reasons are researcher- or hospital-related logistical reasons such as no consulting room available or due to different hospital locations. No time means patient had no time; too ill or too old means patient thinks he or she is too ill or too old to participate.

GHZ, Groene Hart Ziekenhuis; HMC, Haaglanden Medical Centre; LUMC, Leiden University Medical Centre; MUMC, Maastricht University Medical Centre.

**Supplementary table 1**. Full list of reasons for hospitalization and their classification

| ID | Reason for hospital admission | All-cause | Acute | IBD-related |
| --- | --- | --- | --- | --- |
| 97 | Anemia | 1 | 1 | 0 |
| 44 | Anemia due to Crohn’s disease activity | 1 | 1 | 1 |
| 55 | Pneumothorax | 1 | 1 | 0 |
|  | Persistent pneumothorax | 1 | 1 | 0 |
| 58 | IBD exacerbation | 1 | 1 | 1 |
|  | Progressive disease | 1 | 1 | 1 |
|  | Progressive disease | 1 | 1 | 1 |
| 74 | Pleural fluid | 1 | 1 | 0 |
| 77 | Dislocation of proximal interphalangeal joint needing surgery | 1 | 1 | 0 |
| 79 | Surgery M. Dupuytren | 1 | 0 | 0 |
| 83 | Phacoemulsification with intraocular lens | 1 | 0 | 0 |
|  | Phacoemulsification with intraocular lens | 1 | 0 | 0 |
| 1 | Aneurysm a. femoralis superficialis needing stent | 1 | 1 | 0 |
| 2 | Transient Ischemic Attack | 1 | 1 | 0 |
| 3 | Inguinal herniation needing surgery | 1 | 0 | 0 |
| 6 | Urinary tract infection | 1 | 1 | 0 |
|  | Urinary tract infection | 1 | 1 | 0 |
| 8 | Influenza-A | 1 | 1 | 0 |
|  | Cataract needing surgery | 1 | 0 | 0 |
| 12 | Subdural hematoma after trauma | 1 | 1 | 0 |
| 13 | Hospital admission abroad; anamnestic ‘ thick feet needing diuretics’ | 1 | 1 | 0 |
| 17 | Fistula needing seton | 1 | 1 | 1 |
| 19 | Cardiology admission for placing constant loop recorder | 1 | 0 | 0 |
|  | Placing pacemaker | 1 | 0 | 0 |
| 23 | Hernia cicatricalis needing surgery | 1 | 0 | 0 |
| 34 | Instable angina pectoris | 1 | 1 | 0 |
|  | Hernia inguinalis needing surgery | 1 | 0 | 0 |
| 36 | Terminal ileitis due to Crohn’s disease needing surgery | 1 | 1 | 1 |
| 38 | Carcinoma of prostate needing surgery | 1 | 0 | 0 |
| 46 | Urothelial carcinoma needing resection | 1 | 1 | 0 |
| 50 | Prothesis of the knee | 1 | 0 | 0 |
| 52 | Prothesis of the knee | 1 | 0 | 0 |
| 103 | Coronary artery bypass graft surgery | 1 | 1 | 0 |
| 104 | Transuretral resection of the prostate | 1 | 0 | 0 |
| 105 | Pulmonary emobolism | 1 | 1 | 0 |
| 107 | Cerebrovascular accident | 1 | 1 | 0 |
| 113 | Asthma | 1 | 1 | 0 |
| 116 | Urosepsis | 1 | 1 | 0 |
| 125 | Infection needing antibiotics | 1 | 1 | 0 |
| 128 | Pneumonia | 1 | 1 | 0 |
| 129 | Admission after infliximab infusion with hyponatraemia, hypertension. | 1 | 1 | 1 |
| 131 | Colectomy due to therapyresistant colitis | 1 | 1 | 1 |
| 134 | Diverticulitis | 1 | 1 | 0 |
| 138 | Occlusion of aortic prosthesis | 1 | 1 | 0 |
| 144 | Bypass surgery (femoro-popliteal) | 1 | 0 | 0 |
| 158 | Cholecystectomy | 1 | 0 | 0 |
| 159 | Hypertensive crisis | 1 | 1 | 0 |
| 163 | Bleeding ostomy | 1 | 1 | 1 |
| 169 | Dehydration due to gastroenteritis | 1 | 1 | 0 |
| 178 | Urosepsis | 1 | 1 | 0 |
| 182 | Suspection of sigmoid volvulus | 1 | 1 | 0 |
| 183 | Incision and drainage of fistula | 1 | 1 | 1 |
| 192 | Ileus | 1 | 1 | 1 |
|  | Ileus | 1 | 1 | 1 |
| 194 | Exacerbation IBD | 1 | 1 | 1 |
| 195 | Ileocoecal resection | 1 | 1 | 1 |
| 203 | Surgery for polyposis nasi | 1 | 0 | 0 |
| 207 | Exacerbation IBD | 1 | 1 | 1 |
|  | Exacerbation IBD | 1 | 1 | 1 |
|  | Exacerbation IBD | 1 | 1 | 1 |
|  | Resection jejunum due to stenosis | 1 | 1 | 1 |
| 224 | Pneumonia | 1 | 1 | 0 |
|  | Exacerbation IBD | 1 | 1 | 1 |
| 225 | Exacerbation COPD with fever needing antibiotics | 1 | 1 | 0 |
|  | Acute kidney failure | 1 | 1 | 0 |
| 228 | Viral infection (not specified) | 1 | 1 | 0 |
| 231 | Ileocecal resection | 1 | 1 | 1 |
| 232 | Cholangitis | 1 | 1 | 0 |
|  | Cholangitis | 1 | 1 | 0 |
|  | Cholangitis | 1 | 1 | 0 |
|  | Cholangitis | 1 | 1 | 0 |
| 235 | Attempted suicide | 1 | 1 | 0 |
|  | Abces near appendix needing drainage and antibiotic treatment | 1 | 1 | 1 |
| 250 | Colectomy with ileo-rectal anastomosis due to colorectal carcinoma | 1 | 1 | 1 |
| 257 | Exacerbation IBD needing ileum resection | 1 | 1 | 1 |
|  | Exacerbation | 1 | 1 | 1 |
| 261 | Pneumonia | 1 | 1 | 0 |
| 263 | Collapse | 1 | 1 | 0 |
| 264 | Admission due to psychiatric cause | 1 | 1 | 0 |
| 270 | Acute hearing loss | 1 | 1 | 0 |
| 272 | Rectal blood loss due to rectal ulcers | 1 | 1 | 1 |
| 273 | Cardioversion | 1 | 0 | 0 |
|  | Cardioversion | 1 | 0 | 0 |
|  | Cardioversion | 1 | 0 | 0 |
| 275 | Nephrolithiasis | 1 | 1 | 0 |
| 277 | Ileocecal resection | 1 | 1 | 1 |
|  | Suspected anastomotic leakage needing resection | 1 | 1 | 1 |
| 292 | Palpitations | 1 | 1 | 0 |
| 306 | Laparoscopic removal adnex | 1 | 0 | 0 |
| 309 | Ileus | 1 | 1 | 1 |
|  | Exacerbation COPD | 1 | 1 | 0 |
| 310 | Exacerbation colitis | 1 | 1 | 1 |
|  | Guillanbarree possibly due to azathiopurine | 1 | 1 | 1 |
| 312 | Benign prostate hypertrophy needing surgery | 1 | 0 | 0 |
| 315 | Atrioventricular block | 1 | 1 | 0 |
| 316 | Infected endovascular aortic prothesis due to fistula | 1 | 1 | 1 |
| 325 | Lumbal stenosis needing surgery | 1 | 0 | 0 |
| 329 | Bleeding ulcer | 1 | 1 | 0 |
|  | Cardiac ablation | 1 | 1 | 0 |
|  | Lower gastrointestinal tract bleeding | 1 | 1 | 0 |
| 340 | Stenosis needing ileocecal resection | 1 | 1 | 1 |
| 342 | Cerebrovascular accident | 1 | 1 | 0 |
|  | Decline in general condition | 1 | 1 | 0 |
| 344 | Cerebrovascular accident | 1 | 1 | 0 |
| 346 | Exacerbation IBD | 1 | 1 | 1 |
| 353 | Nissen fundoplication | 1 | 0 | 0 |
|  | Pericarditis | 1 | 1 | 0 |
|  | Chest pain | 1 | 1 | 0 |
| 355 | Palpitations | 1 | 1 | 0 |
|  | Coronary angiogram | 1 | 0 | 0 |
| 358 | Inguinal hernia needing surgery | 1 | 0 | 0 |
| 359 | Cholangitis | 1 | 1 | 0 |
|  | Cataract needing surgery | 1 | 0 | 0 |
| 361 | Fascia dehiscence after surgery | 1 | 1 | 1 |
|  | Abcess near flexura hepatica | 1 | 1 | 1 |
|  | Symptomatic cholecystolithiasis | 1 | 1 | 0 |
|  | Exacerbation IBD | 1 | 1 | 1 |
|  | Enterocutaneous fistula | 1 | 1 | 1 |
| 363 | Resection of rectum and placing ileostoma | 1 | 1 | 1 |
| 365 | Exacerbation IBD | 1 | 1 | 1 |
|  | Exacerbation IBD | 1 | 1 | 1 |
| 367 | Cataract needing surgery | 1 | 0 | 0 |
| 368 | Varicose vein surgery | 1 | 0 | 0 |
|  | Knee prothesis | 1 | 0 | 0 |
| 371 | Rectal blood loss ( ulcerations due to platelet inhibitor use) | 1 | 1 | 0 |
| 375 | Cerebrovascular accident | 1 | 1 | 0 |
|  | Atrium fibrillation | 1 | 1 | 0 |
| 376 | Cerebrovascular accident | 1 | 1 | 0 |
| 381 | Hernia inguinalis | 1 | 0 | 0 |
|  | Prostate carcinoma needing surgery | 1 | 0 | 0 |
| 383 | Kidney stone removal | 1 | 0 | 0 |
| 388 | Progressive anemia | 1 | 1 | 0 |
| 391 | Drainage of fistula | 1 | 1 | 1 |
| 394 | Coronary angiogram | 1 | 0 | 0 |
| 405 | Adrenal crisis | 1 | 1 | 0 |
|  | Urinary tract infection | 1 | 1 | 0 |

**Supplementary table 2.** Full list of infections (any infection and infection needing hospital admission

| ID | Infection type | Any infection | Infection needing hospital admission |
| --- | --- | --- | --- |
| 90 | Pneumonia | 1 | 0 |
| 93 | COVID-19 | 1 | 0 |
| 46 | Pneumonia | 1 | 0 |
| 53 | Pneumonia | 1 | 0 |
| 59 | Undefined infection needing antibiotics | 1 | 0 |
| 64 | Urinary tract infection | 1 | 0 |
| 65 | COVID-19 | 1 | 0 |
| 4 | Gastritis, H. Pylori | 1 | 0 |
| 6 | Urinary tract infection | 1 | 1 |
|  | Urinary tract infection | 1 | 1 |
|  | Gastritis, H. Pylori | 1 | 0 |
| 7 | COVID-19 | 1 | 0 |
| 8 | Influenza-A | 1 | 1 |
| 11 | COVID-19 | 1 | 0 |
| 100 | Mandibular infection | 1 | 0 |
| 108 | Herpes zoster | 1 | 0 |
| 111 | Urinary tract infection | 1 | 0 |
|  | Bronchitis | 1 | 0 |
| 114 | Pneumonia | 1 | 0 |
| 116 | Urosepsis | 1 | 1 |
| 119 | Pneumonia | 1 | 0 |
| 125 | Infection needing antibiotics | 1 | 1 |
| 127 | Pneumonia | 1 | 0 |
| 128 | Pneumonia | 1 | 1 |
| 133 | Urinary tract infection | 1 | 0 |
| 134 | Urinary tract infection | 1 | 0 |
| 139 | Pneumonia | 1 | 0 |
|  | Pneumonia | 1 | 0 |
| 140 | Clostridium difficile | 1 | 0 |
| 143 | Pneumonia | 1 | 0 |
| 146 | Urinary tract infection | 1 | 0 |
| 147 | Bilateral pneumonia | 1 | 0 |
| 148 | Lower respiratory tract infection | 1 | 0 |
|  | Lower respiratory tract infection | 1 | 0 |
| 152 | Campylobacter jejuni enteritis | 1 | 0 |
| 156 | Urinary tract infection | 1 | 0 |
|  | Pneumonia | 1 | 0 |
| 159 | H. Pylori infection | 1 | 0 |
| 160 | Viral infection (not specified) | 1 | 0 |
| 163 | Candida infection | 1 | 0 |
| 164 | Pneumonia | 1 | 0 |
| 165 | Viral infection (not specified) | 1 | 0 |
| 169 | Dehydration due to gastroenteritis | 1 | 1 |
| 171 | Herpes simplex | 1 | 0 |
| 178 | Urosepsis | 1 | 1 |
| 179 | Epididymitis | 1 | 0 |
|  | Pneumonia | 1 | 0 |
|  | Mandibular infection | 1 | 0 |
| 188 | Erysipelas | 1 | 0 |
| 195 | Infection of the skin | 1 | 0 |
|  | Infection of the skin | 1 | 0 |
| 197 | Viral infection (not specified) | 1 | 0 |
| 199 | Pneumonia | 1 | 0 |
| 200 | Herpes zoster | 1 | 0 |
|  | Erythema migrans | 1 | 0 |
| 206 | Viral infection (not specified) | 1 | 0 |
| 208 | Viral infection (not specified) | 1 | 0 |
| 209 | Conjunctivitis | 1 | 0 |
| 210 | Bacterial infection (not specified) | 1 | 0 |
| 211 | Viral infection (not specified) | 1 | 0 |
| 213 | Herpes zoster | 1 | 0 |
| 219 | Blefaritis | 1 | 0 |
| 224 | Pneumonia | 1 | 1 |
|  | Campylobacter infection | 1 | 0 |
| 225 | Urinary tract infection | 1 | 0 |
|  | Exacerbation COPD with fever needing antibiotics | 1 | 1 |
| 226 | Upper respiratory tract infection | 1 | 0 |
| 227 | Upper respiratory tract infection | 1 | 0 |
| 228 | Viral infection (not specified) | 1 | 1 |
| 234 | Viral infection (not specified) | 1 | 0 |
| 236 | Viral infection (not specified) | 1 | 0 |
|  | Viral infection (not specified) | 1 | 0 |
| 238 | Viral infection (not specified) | 1 | 0 |
| 256 | Mandibular infection | 1 | 0 |
| 257 | Bacterial infection | 1 | 0 |
| 261 | Pneumonia | 1 | 1 |
| 280 | Urinary tract infection | 1 | 0 |
| 292 | Campylobacter infection | 1 | 0 |
| 294 | Urinary tract infection | 1 | 0 |
|  | Pneumonia | 1 | 0 |
| 304 | Pneumonia | 1 | 0 |
| 312 | Upper respiratory tract infection | 1 | 0 |
| 327 | Pneumonia | 1 | 0 |
| 329 | Wound infection | 1 | 0 |
| 331 | Bacterial infection | 1 | 0 |
| 340 | Viral infection | 1 | 0 |
| 342 | Viral infection | 1 | 0 |
| 353 | Pericarditis | 1 | 1 |
|  | Rotavirus | 1 | 0 |
| 362 | Gastroenteritis | 1 | 0 |
|  | Upper respiratory tract infection | 1 | 0 |
|  | Pneumonia | 1 | 0 |
| 364 | Skin infection | 1 | 0 |
|  | Infection | 1 | 0 |
|  | Skin infection | 1 | 0 |
| 366 | Viral infection (not specified) | 1 | 0 |
| 372 | Pneumonia | 1 | 0 |
| 373 | Respiratory tract infection | 1 | 0 |
| 374 | Respiratory tract infection | 1 | 0 |
| 375 | Urinary tract infection | 1 | 0 |
| 376 | Pneumonia | 1 | 0 |
| 377 | Urinary tract infection | 1 | 0 |
|  | Urinary tract infection | 1 | 0 |
| 388 | Influenza | 1 | 0 |
| 395 | Urinary tract infection | 1 | 0 |
| 405 | Urinary tract infection | 1 | 1 |

**Supplementary table 3**. Full list of malignancies

| ID | Malignancy type |
| --- | --- |
| 71 | Lung carcinoma |
| 74 | Lung carcinoma with malignant pleural effusion |
| 20 | Oesophageal carcinoma |
| 104 | Adenocarcinoma of the prostate |
| 46 | Urothelial cell carcinoma |
| 147 | Neuroendocrine tumor |
| 174 | Pancreas carcinoma with liver metastasis |
| 177 | Melanoma |
| 195 | Non-melanoma skin cancer |
| 213 | Colorectal carcinoma |
| 311 | Multiple myeloma |
| 250 | Colorectal carcinoma |
| 350 | Lung carcinoma |
| 381 | Adenocarcinoma of the prostate |
| 386 | Lung carcinoma |

**Supplementary table 4.** Mortality causes

| ID | Mortality cause |
| --- | --- |
| 74 | Lung carcinoma, with malignant pleural effusion |
| 113 | Pulmonary comorbidity |
| 23 | Unknown |
| 20 | Oesophageal carcinoma |
| 106 | Extensive arterial vascular disease |
| 145 | Unknown |
| 190 | Infection, not specified |
| 348 | End stage renal disease |
| 370 | Cholangiocarcinoma |
| 386 | Lung carcinoma |
| 398 | Unknown |

**Supplementary table 5.** Types of biologicals used at baseline

|  | Total cohort (n=405) | No risk of frailty (n=207) | Risk of frailty (n=196) | p-value |
| --- | --- | --- | --- | --- |
| Biological | 107 | 52 | 54 | .580 |
| Adalimumab  Infliximab  Ustekinumab  Vedolizumab | 29 (27.1%) 53 (49.5%) 7 (6.5%) 18 (16.8%) | 18 (34.6%) 21 (40.4%) 4 (7.7%) 9 (17.3%) | 11 (20.4%) 31 (57.4%) 3 (5.6%) 9 (16.7%) | .100 .080 .713 .930 |
| Percentages of all biologicals were noted, risk of frailty was measured by G8 questionnaire | | | | |
